# Obituary

**Published:** 1882-02

**Authors:** 


### OBITUARY.

The many friends of Dr. M. S. Dean, one of Chicago's oldest and most esteemed practitioners of dentistry, will be shocked and pained to learn of his sudden death which took place Saturday morning, January 28th, at his room, No. 34 Monroe street. He had not been feeling as well as usual for some days, but had attended to his professional duties Friday, and spent the evening with friends. He was found at 8 o'clock, A. M., apparently in sweet sleep, but it proved to be the sleep of death. The eyes were closed, the face calm and pleasant in expression, and he had evidently never wakened from his natural sleep. A physician was hastily summoned, but life was extinct, though the body was still warm.

Dr. Dean was 57 years of age. He was a native of Vermont, and came West while still a youth, and passed the earlier years of his practice in Dundas, Canada, and Marshall, Mich., whence he removed to Chicago in 1864. In the time since then he had not only built up an extensive practice, but had won a host of warm friends, all of whom will mourn him as a brother lost. He always manifested a deep interest in his chosen profession, and took a prominent part in every advance movement. He was honored with the highest offices in the city, State and national dental associations which it was in the power of his profession to bestow. He was a man of fine literary attainments—a close student, who studied for the love of study. Numerous and valuable papers in the transactions of the American Dental Association and the Illinois Dental Society attest the value and originality of his contributions to the literature of the dental profession, and his translation of the French work of Lagros and Magitot on the Dental Follicle was accompanied by an introduction embodying his own original investigations and discoveries, which make that work a valuable contribution to the permanent literature of the profession and a positive addition to the sum of human knowledge on that subject. Dr. Dean was possessed of a keen sense of justice, and was strictly upright in all his dealings with his fellow-men. In his death the profession loses one of its most valued members, and many a household will feel that it has suffered a personal bereavement.

